# Where Should I Send It? Optimizing the Submission Decision Process

**DOI:** 10.1371/journal.pone.0115451

**Published:** 2015-01-23

**Authors:** Santiago Salinas, Stephan B. Munch

**Affiliations:** 1 Center for Stock Assessment Research, University of California Santa Cruz, Santa Cruz, California, United States of America; 2 Southwest Fisheries Science Center, National Oceanic and Atmospheric Administration, Santa Cruz, California, United States of America; University of Namur, BELGIUM

## Abstract

How do scientists decide where to submit manuscripts? Many factors influence this decision, including prestige, acceptance probability, turnaround time, target audience, fit, and impact factor. Here, we present a framework for evaluating where to submit a manuscript based on the theory of Markov decision processes. We derive two models, one in which an author is trying to optimally maximize citations and another in which that goal is balanced by either minimizing the number of resubmissions or the total time in review. We parameterize the models with data on acceptance probability, submission-to-decision times, and impact factors for 61 ecology journals. We find that submission sequences beginning with *Ecology Letters*, *Ecological Monographs*, or *PLOS ONE* could be optimal depending on the importance given to time to acceptance or number of resubmissions. This analysis provides some guidance on where to submit a manuscript given the individual-specific values assigned to these disparate objectives.

## Introduction

The question of where to submit a finished manuscript is an inescapable part of life as a scientist. Yet, it has received little attention beyond cursory advice in “how to be a scientist” guides. Many factors can drive this decision, including journal prestige, likelihood of acceptance, turnaround time, target audience, and impact factor (IF).

Journal impact factors (IF) are now widely used by universities and agencies to evaluate performance and make hiring and funding decisions [1, 2]. Moreover—and despite the many arguments against the IF (e.g., [3–5])—it is undeniable that scientists are well aware of, and make submission decisions based on, them. 85.6% of 1,250 ecologists indicated that a high journal IF is a ‘very important’ to ‘important’ criterion in selecting where to send manuscripts [6], data on resubmission patterns suggest a flow from higher to lower IF journals [7], and a survey of Canadian researchers found that journal prestige and impact factor greatly outranked other criteria [8].

Although a poor predictor of the ultimate success of any particular paper [9], the journal impact factor offers a concise summary of the expected citation rate for papers published therein. Assuming that scientists would like to have their publications cited, the IF therefore is a potentially useful criterion in deciding where to send their work (roughly ¼ of all natural science and engineering articles go uncited in the first 5 years of publication [10]). Thus, a researcher seeking to maximize citation counts might choose to submit to the journal with the highest IF and then work down the IF list as the manuscript is rejected. From a decision theoretic point of view, this is known as a ‘myopic policy’; it ignores the value of subsequent actions and opportunity costs (e.g., what happens if the paper is rejected). In fact, many other factors are relevant to this decision, including likelihood of acceptance, the time to publication, and the appropriateness of the target audience. Here, we present a simple framework for evaluating where to submit a manuscript based on the theory of Markov decision processes [11] and apply it to journals publishing ecological research. We also use this framework to evaluate trade-offs between citations and competing concerns such as the time to publication and number of revisions. We compare the follow-the-IF strategy to the one we obtain and arrive at some surprising conclusions.

## Methods

We approach this problem in two ways. First, we derive the optimal submission sequence for a scientist attempting to maximize the expected number of citations obtained over some finite period. Recognizing that revising and resubmitting a manuscript multiple times is both time-consuming and demoralizing, in our second approach we solve the dual-objective problem posed by balancing the trade-off between the expected number of citations and either the expected number of revisions or the time to publication.

### Model 1. Maximizing citations

In this first model, we assume that a scientist is trying to maximize the expected number of citations his/her manuscript receives over a finite time interval ending at time *T*. For example, *T* might be the time to tenure for a new professor, the time to retirement for a midcareer scientist, or the researcher’s life expectancy. Let *C* denote the expected number of citations accruing to a manuscript over [0,*T*] given the sequence of journals to which the paper is submitted.

If the paper is certain to be accepted by the first journal, then *C* is simply the product of the expected citation rate for papers in that journal and the time remaining once the publication is in print. The expected number of citations per year is approximately the impact factor of a journal, since the IF is a “measure of the frequency with which the ‘average article’ in a journal has been cited” over a 1-yr period [12].

However, it is typically not certain that a publication will be accepted. Thus, we must account for the possibility of rejection, followed by a round of reformatting and revision, followed by submission to the next journal, etc. During this time, there is, of course, some chance that an analogous publication will be produced by a competing author, i.e., you get scooped. For simplicity, we assume that a publication that has been scooped has negligible value. Given these criteria, the expected number of citations, *C*, is

$$C=q^{-1}{\sum_{j=1}^{N}\alpha_{j}\lambda_{j}\left[T-\sum_{k=1}^{j}\tau_{j}-\left(j-1\right)t_{R}\right]}^{+}\prod_{k=1}^{j-1}\left(1-\alpha_{k}\right){\left(1-s\right)}^{\tau_{k}+t_{R}}$$ (1)

where *α_j_* is the acceptance rate of journal *j*, *λ_j_* is the expected number of citations for a paper in that journal, *τ_j_* is the time (days) from submission to publication, *t_R_* (days) is the revision time, and *s* is the daily probability of getting scooped by other researchers (see S1 Model for model details). The term in square brackets is the time remaining over which citations can be accumulated with the superscript + indicating that negative values are replaced by 0. The product term indicates the probability of having neither been accepted nor scooped prior to submission to the j^th^ journal and the quantity *q* is the normalization constant. Here, we have assumed that the average rate at which citations are accumulated is constant and is roughly equal to the journal’s impact factor.

The goal is to maximize *C* over possible submission sequences. As detailed in S1 Model, the criteria for choosing to start with journal *j* over journal *k* is

$$\begin{array}{l} \alpha_{j}\lambda_{j}\left\{T-\tau_{j}-\left(1-\alpha_{k}\right){\left(1-s\right)}^{t_{R}+\tau_{k}}\alpha_{k}\lambda_{k}\left(T-\tau_{k}\right)+\left(1-\alpha_{k}\right){\left(1-s\right)}^{t_{R}+\tau_{k}}\alpha_{j}\lambda_{j}\left(T-\tau_{k}-t_{R}-\tau_{j}\right)\right\}\geq \\ \alpha_{k}\lambda_{k}\left\{T-\tau_{k}-\left(1-\alpha_{j}\right){\left(1-s\right)}^{t_{R}+\tau_{j}}\alpha_{j}\lambda_{j}\left(T-\tau_{j}\right)+\left(1-\alpha_{j}\right){\left(1-s\right)}^{t_{R}+\tau_{j}}\alpha_{k}\lambda_{k}\left(T-\tau_{j}-t_{R}-\tau_{k}\right)\right\} \end{array}$$ (2)

Criterion (2) is applied to all pairs of journals and the optimal journal is the one that dominates the greatest number of other journals. Note that this criterion merely establishes which journal to visit first. To determine an optimal submission schedule, one starts with T, evaluates all journals, finds the best journal, j*, then reduces *T* by τ_j*_ + *t_R_* and starts over with j* removed from the list.

This ranking scheme is dependent on the set of journals being evaluated. In cases where *T* is large relative to the publication and revision times (i.e., $\frac{\tau_{k}+t_{R}}{T-\tau_{j}}\approx 0$), we can simplify this inequality and refine our ranking of journals based on an index value *V_j_* given by

$$V_{j}=\frac{\alpha_{j}\lambda_{j}\left(1-\tau_{j}/T\right)}{1-\left(1-\frac{\tau_{j}}{T}-\frac{t_{R}}{T}\right)\left(1-\alpha_{j}\right){\left(1-s\right)}^{t_{R}+\tau_{j}}}$$ (3)

(see the Supporting Information for a derivation). This allows us to give each journal an independent score without evaluating all pairwise comparisons and is independent of the pool of journals being compared.

Note that we have implicitly assumed that all manuscripts are of equal relevance to all journals. This is clearly not the case. The journals under ISI’s ‘Ecology’ category are still quite heterogeneous—a single manuscript is unlikely to be relevant to both *Polar Biology* and *Biotropica*. When considering where to send a specific manuscript, the ranking criteria (2 or 3) should be applied to the relevant subset of journals.

### Model 2. Balancing citations and frustrations

Obviously, there are factors beyond likely citation rates that authors might value in choosing where to submit their manuscript. Here, we consider that authors might also want to minimize either the number of rejections and resubmissions or the total time it takes for their paper to get accepted. Following a given submission sequence, the expected number of submissions is given by

$$R=q^{-1}\sum_{j=1}^{N}j\alpha_{j}\prod_{i=1}^{j-1}\left(1-\alpha_{i}\right){\left(1-s\right)}^{t_{R}+\tau_{i}}\text{H}\left(T-\sum_{k=1}^{j}\tau_{j}-\left(j-1\right)t_{R}\right)$$ (4)

Here, H is the Heaviside function, taking the value 1 if its argument is positive and zero otherwise. This is used to ensure that the expected number of resubmissions is calculated within the time horizon *T*.

Since individual authors are expected to vary in how much weight they assign to each criterion, we adopt a multi-objective programming approach [13, 14]. That is, we sought to identify the boundary where the expected number of resubmissions is minimized for a given number of citations. Although algorithms for finding the ‘efficiency frontier’ exist (see, e.g., [15, 13]), the space to be evaluated is vast (between 1.4x10^17^ and 4x10^76^depending on the algorithm employed). We therefore adopted a Monte Carlo approach. We used a Metropolis algorithm in which a proposed submission sequence was obtained from the current ‘best’ sequence by swapping the position of two journals at random. For both the current and new sequence, we calculated *C* and *R* and replaced the current sequence with probability determined by the ratios *C_proposed_*/*C_current_* and *R_current_*/*R_proposed_*. To ensure that the space of possible orderings was well covered, we used multiple starting conditions representing all N(N-1) possible pairs of first two journals. From these simulations, we obtained *C* and *R* for 3.2 million different submission schedules. We identified the efficiency frontier by finding the sequence that minimized *R* for each value of *C*. For these analyses, we assumed that *T* = 5 years, *s* = 0.001, and *t_r_* = 30 days.

Using an analogous approach, we also evaluated the trade-off between the expected number of citations and the mean time to acceptance. The mean time to acceptance is given by

$$P=q^{-1}\sum_{j=1}^{N}\left[\tau_{i}+\left(j-1\right)t_{R}\right]\alpha_{j}\prod_{i=1}^{j-1}\left(1-\alpha_{i}\right){\left(1-s\right)}^{t_{R}+\tau_{i}}\text{H}\left(T-\sum_{k=1}^{j}\tau_{j}-\left(j-1\right)t_{R}\right)$$ (5)

### Data

To gather data on acceptance probability and submission-to-decision times, we contacted the editor or managing editor of each journal listed under the ‘Ecology’ category in ISI’s Web of Science (n = 125) as well as 6 general journals that publish ecological research (*Nature*, *Nature Climate Change*, *PLOS ONE*, *Proceedings of the Royal Society B*, *Proceedings of the National Academy of Sciences*, and *Science*). We excluded from analysis journals that clearly do not follow the standard publishing model (e.g., *Annual Review of Ecology, Evolution, and Systematics* has an acceptance rate of 90% because it is an invitation-only journal). We obtained all necessary data from 61 journals (47% response; S1 Table). Journal metrics were gathered from ISI’s Journal Citation Reports. Our dataset includes journals from the entire IF spectrum (IF of responding and non-responding journals were similar; S1 Fig.). Unfortunately, some premier journals are missing from our analysis due to a lack of response. Particularly noteworthy is the absence of *PLOS Biology*, despite its core objective “to pursue a publishing strategy that optimizes the openness, quality and integrity of the publication process.”

## Results

**Figure 1:**
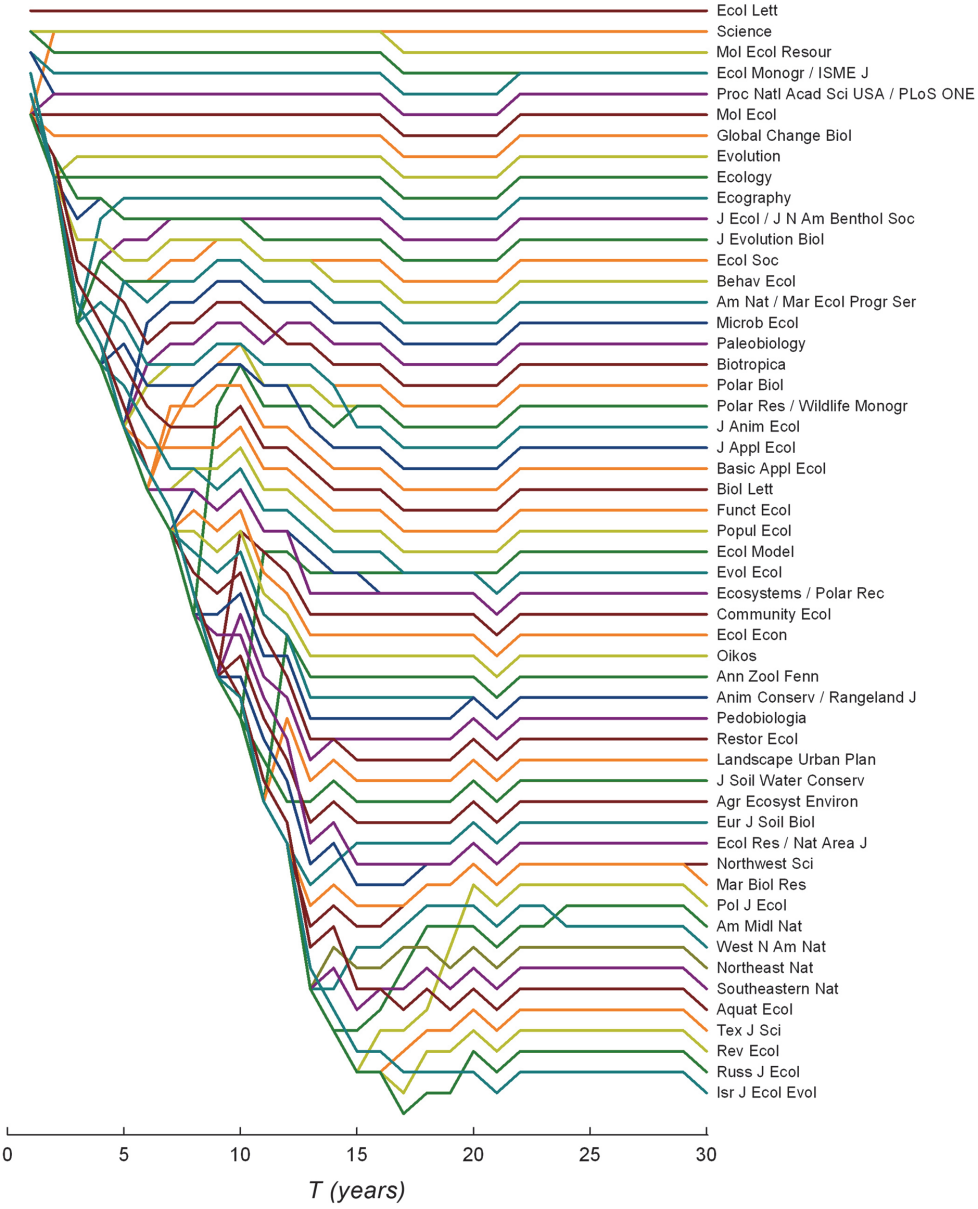
Ranking of journals based solely on maximizing citations over different periods of time, *T* (i.e., maximizing *C* in eq. 1 over all possible submission strategies; probability of getting scooped, *s* = 0.001, time for revisions after each submission, *t* *_R_* = 30 days). The time over which to accrue citations, *T*, changes the optimal submission ranking considerably.

Assuming the probability of getting scooped is relatively low (*s* =0.001), *Ecology Letters* is the optimal ecological journal for any *T*. However, for other journals, the time over which to accrue citations, *T*, changes the optimal submission ranking considerably (Fig. 1). The shape of Fig. 1 is due to the fact that the number of journals that can be tried increases with *T*. Submitting to every single one of the 61 journals would take ~15 years (and a very thick skin). When *T* is small, relatively few journals can be tried and these are all high-ranking. A higher risk of being scooped (*s* = 0.01) pushes *Molecular Ecology Resources* to share the top optimal spot with *Ecology Letters*; the top 10 journals remain the same but in slightly different order (S2 Fig.).

**Figure 2:**
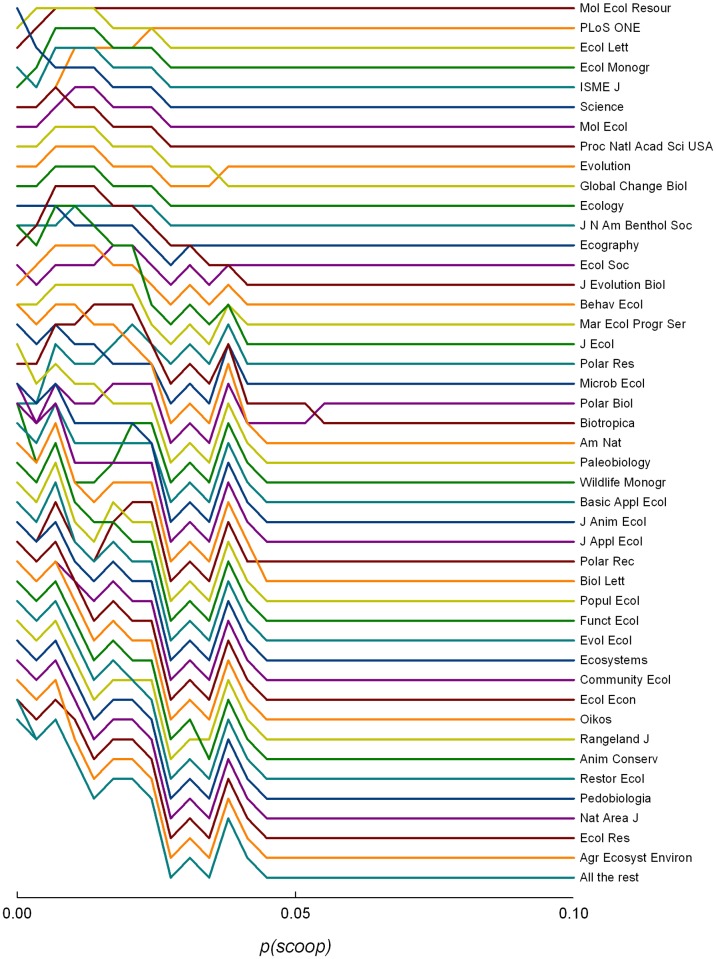
Ranking of journals, under a citation maximization framework, for different values of scooping probability, *s* (*T* = 10 years, *t* *_R_* = 30 days).

For an intermediate *T* (10 years), *Molecular Ecology Resources* becomes the top-ranked journal for most values of *s*, followed by *PLOS ONE* and *Ecology Letters* (Fig. 2). The same general patterns hold at *T* = 2 years (S3 Fig.). Revision times had very little effect on overall rankings.

Scoring journals using Eq. 3 allowed us to avoid pairwise comparisons and produced rankings that are quite similar to those obtained from the full model (Spearman’s ρ = 0.920, p < 0.001). Thus, it is possible to extend this analysis to other journals without re-evaluating all pairs if acceptance rate and submission-to-decision times are known.

If we are trying to maximize citations and allow for the fact that a manuscript might be rejected, the strategy of just working down the IF ranking is clearly suboptimal. But how bad is it, exactly? When considering all ecological journals, following the IF ranking produces an expected number of citations that is never less than 90% of that obtained following the optimal submission schedule, whenever *T* is >3 yrs. However, if only a subject-specific subset of journals was used (e.g., only journals that would realistically accept a modeling paper like this one), the citations resulting from using the IF ranking are relatively worse, but still within ~70% of optimal. Thus, if an author cares only about accumulating citations and wishes not to do any calculating, working down the IF list is not too bad of a strategy.

**Figure 3:**
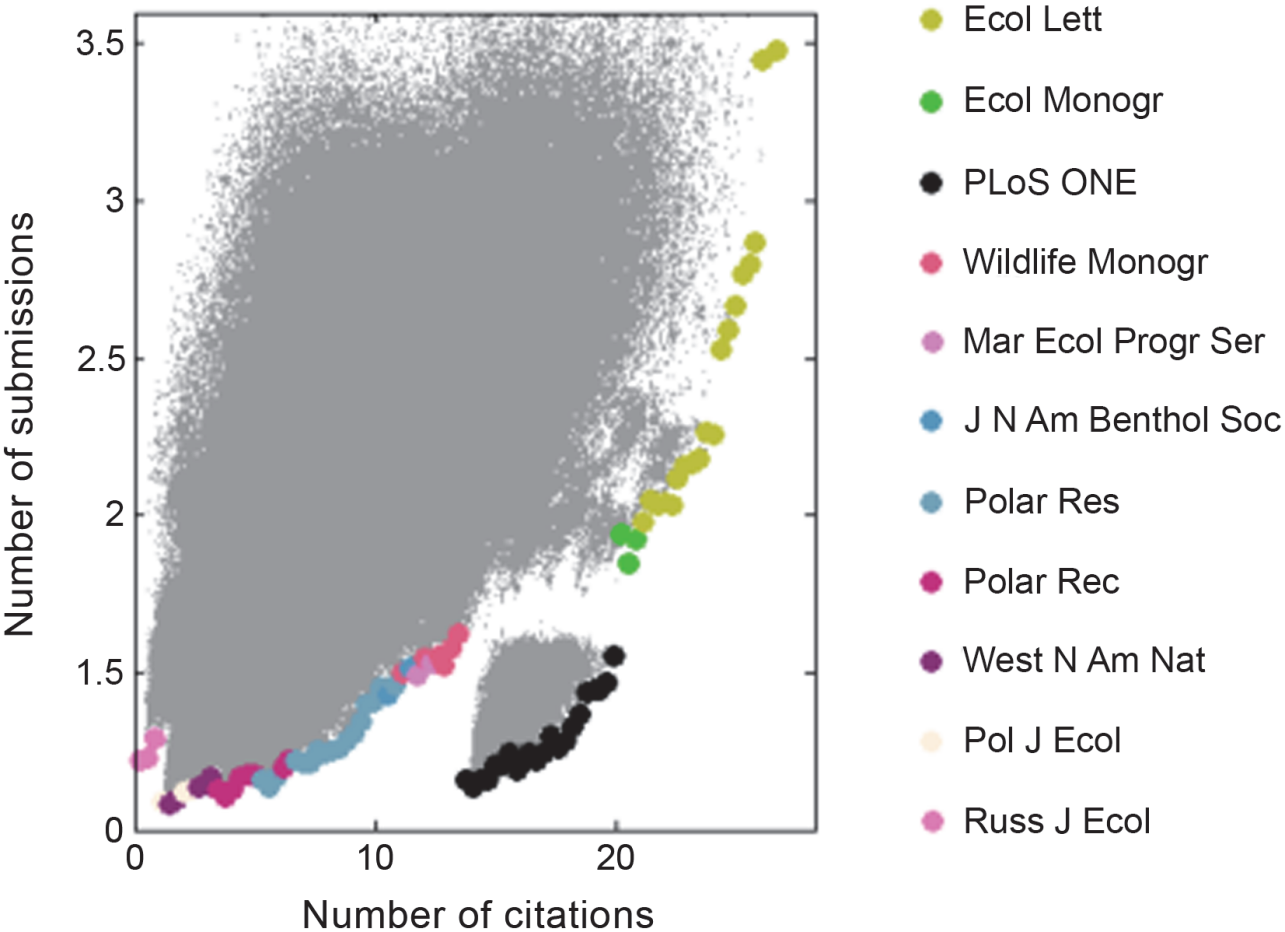
3,200,000 different submission strategies (each grey dot) are evaluated in terms of expected number of citations (over 5 years) and number of submissions needed before acceptance (*s* = 0.002, *t* *_R_* = 30 days). Highlighted are the top journals for citation-maximizing strategies that minimize resubmissions (efficiency frontier). *Ecology Letters* dominates the high expected number of citations area, while *PLOS ONE* is the clear optimal choice at intermediate citations.

**Figure 4:**
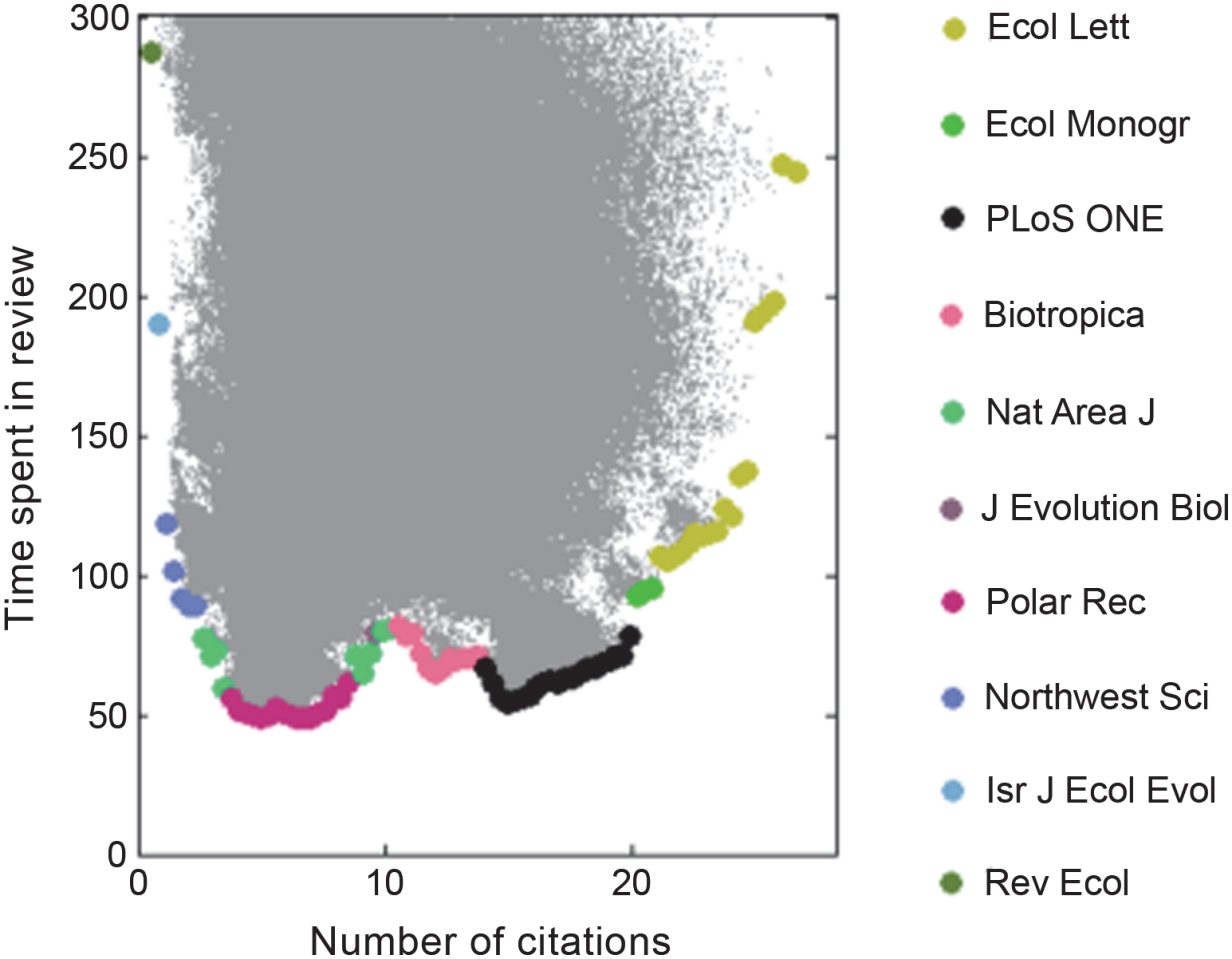
Expected number of citations (over 5 years) and time spent in review for 3,200,000 different submission strategies (*s* = 0.002, *t* *_R_* = 30 days). Highlighted are the top journals for citation-maximizing strategies that minimize time spent in review.

However, researchers may not wish to go through multiple resubmissions or might want to minimize the time it takes for their paper to appear in print. In light of this, we evaluated the trade-off between the expected number of citations and the expected number of times the manuscript will be submitted (Fig. 3 & S4 Fig.) and the mean time to decision (Fig. 4 & S5 Fig.). Note that in both cases, the efficiency frontier is nonlinear and has a complex structure resulting from the discrete nature of the state space. These figures suggest that three tiers exist: the top citation-getters (*Ecology Letters*, *Science*, *Ecological Monographs*, *ISME Journal*), the oddly high-value *PLOS ONE*, and the more specialized/focused journals. In keeping with the results from model 1, the simulation indicates that sequences begun with *Ecology Letters* obtain the highest expected number of citations. However, authors willing to give up four to fourteen citations on average can save themselves up to 0.5 to 1.5 revisions (Fig. 3) and between 30 and 150 days (Fig. 4) by submitting first to *PLOS ONE.* Indeed, the sharp nonlinearity indicates that publishing in any of the journals to the left of *PLOS ONE* would result in lower citations for a negligible reduction in resubmissions or publication time.

## Discussion

Given the negative relationship between IF and the probability of acceptance (S1 Table, [6]), we expected the follow-the-IF strategy to be sub-optimal. Quite surprisingly, following an IF-ranked submission strategy was negligibly worse than the optimal strategy, which included important considerations like time over which to maximize the citation count and the inherent risks of trying new journals (e.g., getting scooped). Following the IF heuristic is quite close to optimal when the chance of getting scooped is low or *T* is large. This result, however, relies on authors’ willingness to re-submit however many times are necessary.

If, in addition to maximizing citations, a scientist is interested in keeping the number of re-submissions low, Fig. 3 should provide some guidance. *PLOS ONE* is a clear winner for low to moderate expected citations (less than 20 in 5 years). At the high end, submission sequences beginning with *Ecology Letters* and *Ecological Monographs* surprisingly provide a better value than those starting with *Science*, the top IF journal in our dataset.

We note that while the model we developed is quite general and could be applied to determine where to publish for any discipline, these specific results apply only to the subset of ecological journals for which we had data. It would be interesting to evaluate whether the ‘IF heuristic’ is nearly optimal in other disciplines, or whether a sharply nonlinear trade-off between citations and ‘frustrations’ is common across disciplines or unique to ecology.

Many important considerations were left out of this analysis. Price per article, for instance, may dissuade some authors from submitting to costly journals (see [16] for an analysis of publication fees and article influence scores). Our model also makes several important simplifications. First, we assumed that the time to publication is roughly constant among papers at a given journal. Although this is not the case [17], we did not have data on variation in the processing time among papers for each journal. Given the nonlinear dependence of our model on publication time, Jensen’s inequality guarantees that—all else being equal—a journal with variable publication times should rank lower than one in which they are constant.

We have also assumed that the acceptance rate and probability of getting scooped are the same for all manuscripts. Manuscripts vary considerably in relevance and quality, even within a single research group, and this is likely to affect the acceptance rate and probability of being scooped. For instance, manuscripts on the spawning periodicity of mummichogs [18] or the recruitment dynamics of bluefish [19] are in little danger of being scooped and hardly likely to be accepted by *Science* or *Ecology Letters*. In applying our strategy to a real manuscript, authors should start at the “appropriate” level for their article (Aarsen and Budden [20] suggest a method for judging the quality of a manuscript).

Perhaps the most important assumptions we made involve the use of IF to measure the expected citation rate of an article. Article-specific citation rates are not constant [21–23] and the average impact factor may be a poor predictor for an individual article over its citation history [9]. Since the expected number of citations depends linearly on the journal-specific citation rate, the mean is sufficient to summarize variation in the average rate among papers within a journal. However, the assumption that there is no variation through time in the rate at which citations are accumulated at a particular journal is more problematic. To compensate for this, our analysis could be extended to include the time-dependent citation profile derived by Wang et al. [9]. Doing so would dramatically increase the complexity of our model and require estimation of the parameter distributions for each ecological journal.

Along similar lines, we assumed that IF ratings were constant over the decision interval. Though this is clearly not the case, forecasting IFs is outside the scope of this note. Particularly relevant to our results is the fact that *PLOS ONE*’s IF has been declining steadily since 2010. Repeating our analysis without *PLOS ONE*, while assuming all other journals remain the same, dramatically changes the shape of the efficiency frontier by eliminating the concavity that appeared in Figs. 3–4 (S6 & S7 Figs.). *Ecological Monographs* and *ISME Journal* largely take the place of *PLOS ONE*, but the absence of a sharp corner to their left means that many other journals could be optimal places to submit, depending on the relative importance of citations, revisions, and publication time.

So, where should you send it? The answer, not surprisingly, is that it depends on the relative importance of citations, revisions, and publication time. This analysis provides some guidance on where to submit a manuscript given the individual-specific values assigned to these disparate objectives. Of course, our analysis was restricted to the subset of journals that were willing to provide data. In order for researchers to evaluate the full range of possibilities we strongly urge journals make acceptance rates and publication times freely available.

## Supporting Information

S1 Fig Impact factor of journals that responded to our request for data did not differ from those that did not respond. (PDF)

S2 Fig Sensitivity of journal ranking to varying values of *T* assuming a relatively high scooping probability (*s* = 0.01). (PDF)

S3 Fig Sensitivity of journal ranking to varying values of *s* assuming a relatively short period of interest for the accumulation of citations (*T* = 2 years). (PDF)

S4 Fig Expected number of citations for a given number of submissions for 3,200,000 different journal ranking combinations. Highlighted are the second-top journals for citation-maximizing strategies that minimize re-submissions (i.e., the journals that follow those in Fig. 3).
(PDF)

S5 Fig Expected number of citations (over 5 years) and time spent in review for 3,200,000 different journal ranking combinations. Highlighted are the second-top journals for citation-maximizing strategies that minimize time spent in review (i.e., the journals that follow those in Fig. 3).
(DOCX)

S6 Fig Expected number of citations (over 5 years) and time spent in review for 3,200,000 different journal rankings excluding *PLOS ONE* from the analysis. Highlighted are the top journals for citation-maximizing strategies that minimize re-submissions.
(PDF)

S7 Fig Expected number of citations (over 5 years) and time spent in review for 3,200,000 different journal rankings excluding *PLOS ONE* from the analysis. Highlighted are the top journals for citation-maximizing strategies that minimize time spent in review.
(DOCX)

S1 Model Model derivation. (PDF)

S1 Table Data used in the analysis. (PDF)

S1 Text Editors who contributed data. (PDF)
