# Supplementary material for: Where Should I Send It? Optimizing the Submission Decision Process
Source: PLoS One. 2015 Jan 23;10(1):e0115451. doi: 10.1371/journal.pone.0115451 (PMC4304711; doi:10.1371/journal.pone.0115451)

Figure S1

Impact factor of journals that responded to our request for data did not differ from those that did not respond

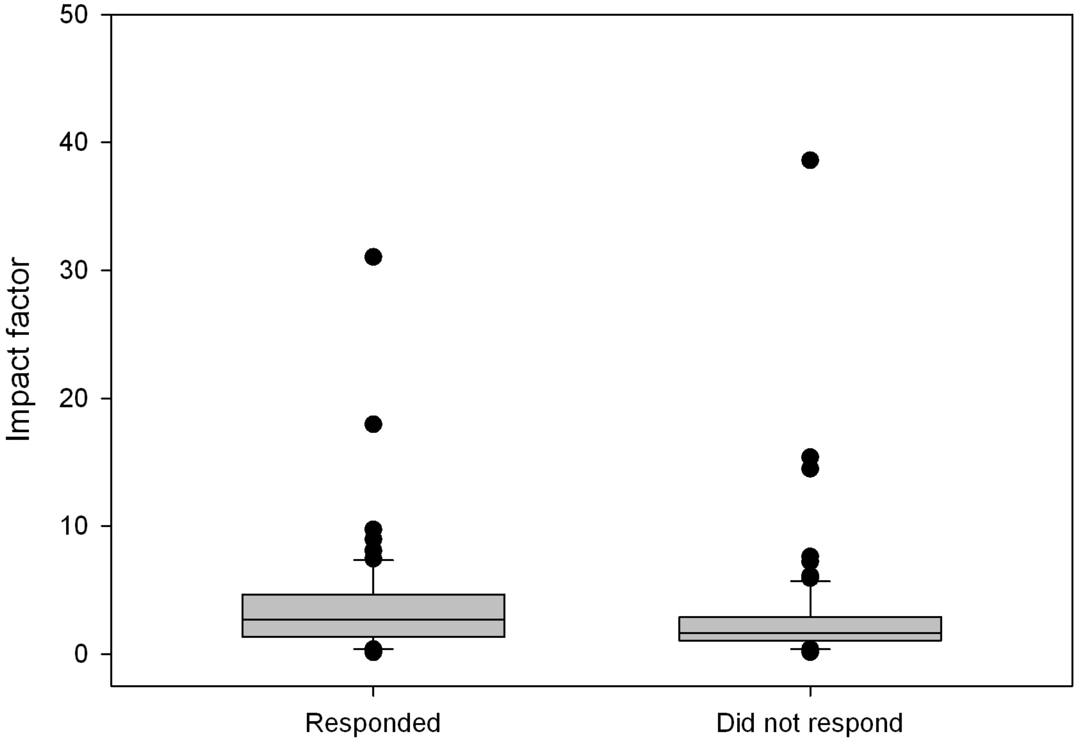

Supplement: S1 Fig — (PDF) [file pone.0115451.s001.pdf]
