# Supplementary material for: Where Should I Send It? Optimizing the Submission Decision Process
Source: PLoS One. 2015 Jan 23;10(1):e0115451. doi: 10.1371/journal.pone.0115451 (PMC4304711; doi:10.1371/journal.pone.0115451)

Figure S3

Sensitivity of journal ranking to varying values of *s* assuming a relatively short period of interest for the accumulation of citations (T = 2 years).

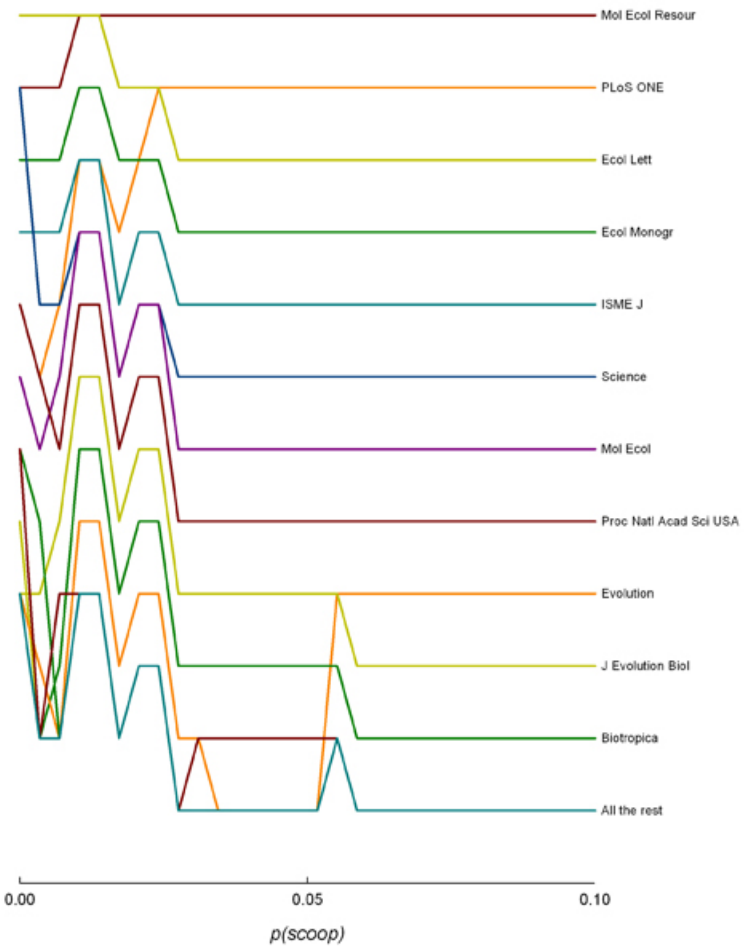

Supplement: S3 Fig — (PDF) [file pone.0115451.s003.pdf]
