# Supplementary material for: Where Should I Send It? Optimizing the Submission Decision Process
Source: PLoS One. 2015 Jan 23;10(1):e0115451. doi: 10.1371/journal.pone.0115451 (PMC4304711; doi:10.1371/journal.pone.0115451)

Figure S4

Expected number of citations for a given number of submissions for 3,200,000 different journal ranking combinations. Highlighted are the second-top journals for citation-maximizing strategies that minimize re-submissions (i.e., the journals that follow those in Figure 3).

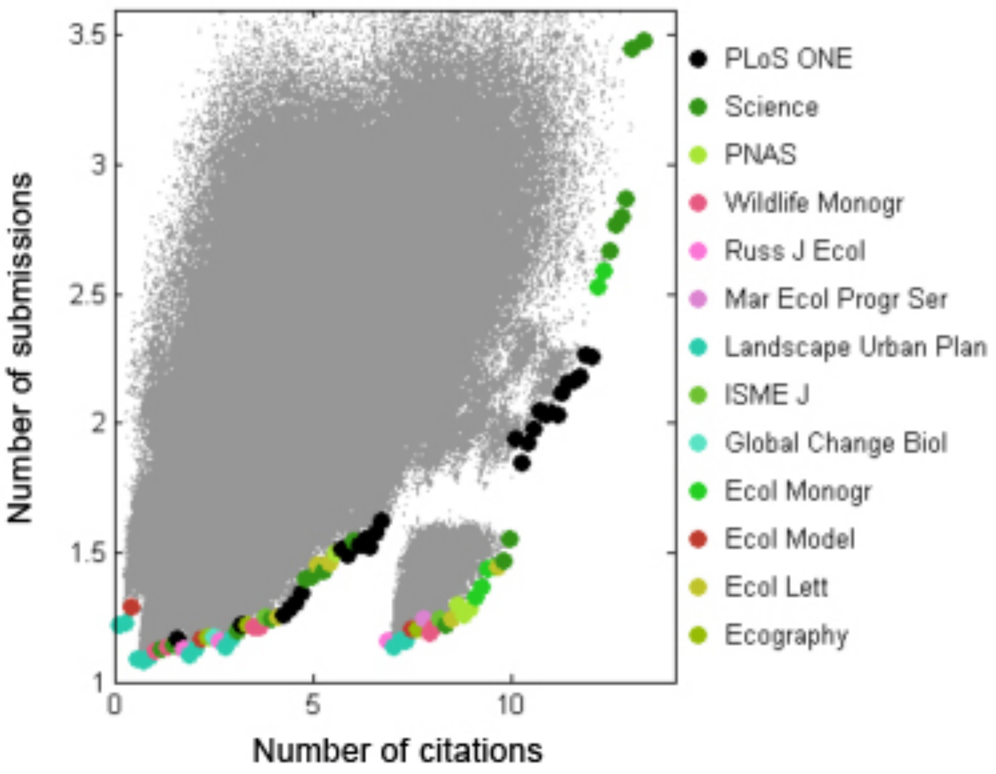

Supplement: S4 Fig — Highlighted are the second-top journals for citation-maximizing strategies that minimize re-submissions (i.e., the journals that follow those in Fig. 3). (PDF) [file pone.0115451.s004.pdf]
