# Supplementary material for: Where Should I Send It? Optimizing the Submission Decision Process
Source: PLoS One. 2015 Jan 23;10(1):e0115451. doi: 10.1371/journal.pone.0115451 (PMC4304711; doi:10.1371/journal.pone.0115451)
Supplement: S5 Fig — Highlighted are the second-top journals for citation-maximizing strategies that minimize time spent in review (i.e., the journals that follow those in Fig. 3). (DOCX) [file pone.0115451.s005.docx]

**Figure S5**

**Expected number of citations (over 5 years) and time spent in review for 3,200,000 different journal ranking combinations. Highlighted are the second-top journals for citation-maximizing strategies that minimize time spent in review (i.e., the journals that follow those in Figure 3).**

**
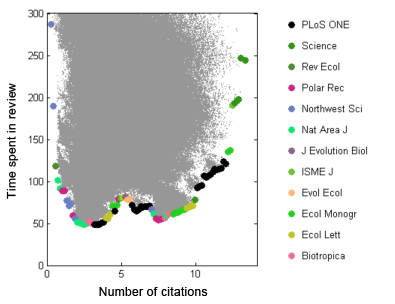
**
