# Supplementary material for: Where Should I Send It? Optimizing the Submission Decision Process
Source: PLoS One. 2015 Jan 23;10(1):e0115451. doi: 10.1371/journal.pone.0115451 (PMC4304711; doi:10.1371/journal.pone.0115451)

Figure S6

Expected number of citations (over 5 years) and time spent in review for 3,200,000 different journal rankings <u>excluding *PLoS ONE* from the analysis</u>. Highlighted are the top journals for citation-maximizing strategies that minimize re-submissions.

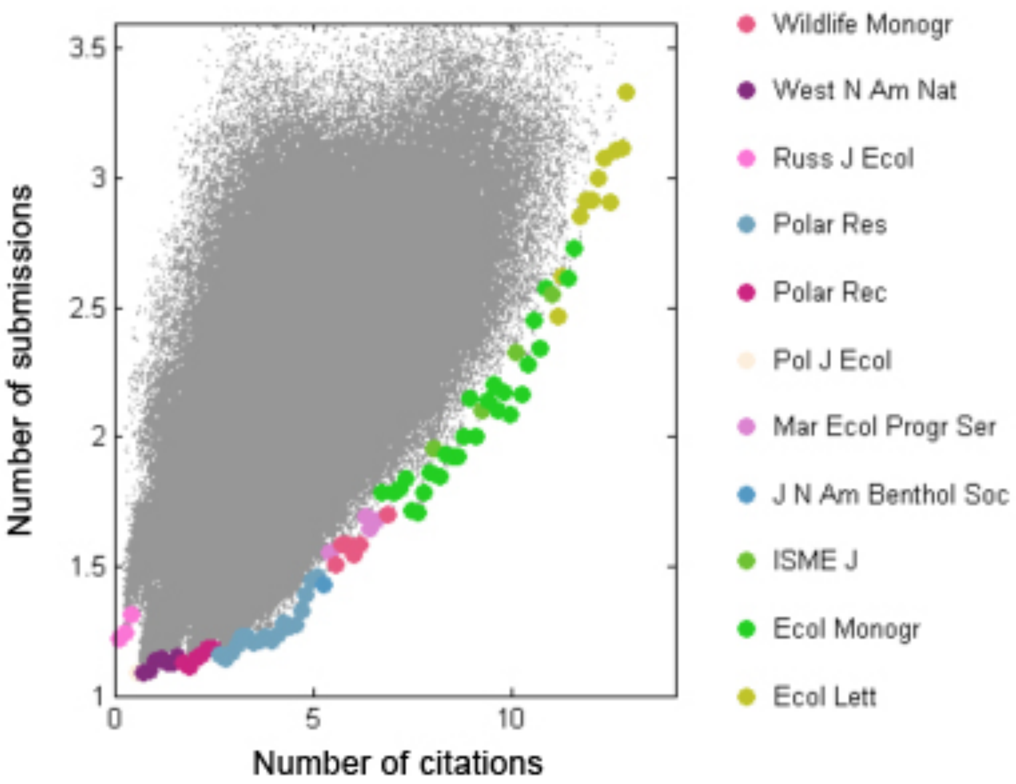

Supplement: S6 Fig — Highlighted are the top journals for citation-maximizing strategies that minimize re-submissions. (PDF) [file pone.0115451.s006.pdf]
