# Supplementary material for: Where Should I Send It? Optimizing the Submission Decision Process
Source: PLoS One. 2015 Jan 23;10(1):e0115451. doi: 10.1371/journal.pone.0115451 (PMC4304711; doi:10.1371/journal.pone.0115451)
Supplement: S7 Fig — Highlighted are the top journals for citation-maximizing strategies that minimize time spent in review. (DOCX) [file pone.0115451.s007.docx]

**Figure S7**

**Expected number of citations (over 5 years) for a given number of submissions for 3,200,000 different journal rankings excluding *PLoS ONE* from the analysis. Highlighted are the top journals for citation-maximizing strategies that minimize time spent in review.**

**
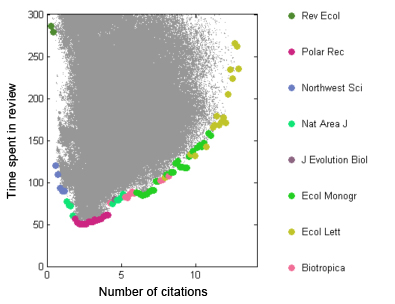
**
