# Supplementary material for: Where Should I Send It? Optimizing the Submission Decision Process
Source: PLoS One. 2015 Jan 23;10(1):e0115451. doi: 10.1371/journal.pone.0115451 (PMC4304711; doi:10.1371/journal.pone.0115451)
Supplement: S1 Model — (PDF) [file pone.0115451.s008.pdf]

Model S1

Model derivation

The idea behind the model is that a scientist is trying to maximize his/her citation count, C, over a given period of time

$$C = \alpha_j \lambda_j (T - \tau_j) + (1 - \alpha_j)(1 - s)^{T - \tau_j - t_r} R$$

where α_j is the acceptance rate of journal *j*, λ_j is its impact factor, τ_j is the time from submission to publication, t_R is the revision time, and *s* is the probability of getting scooped by other researchers. *R* indicates the remainder of the expression obtained by re-starting from journal *k*, *l*, etc. The goal is to maximize *C* by deciding the sequence of journals to try.

Let C_{jk} indicate the expected citation count starting from journal j, then going to k, and continuing optimally from there. To evaluate this expression, we expand R to make explicit the dependence on the second journal, and obtain

$$C_{jk} = \alpha_j \lambda_j (T - \tau_j) + (1 - \alpha_j)(1 - s)^{t_R + \tau_j} \{ \alpha_k \lambda_k (T - \tau_j - t_R - \tau_k) + (1 - \alpha_k)(1 - s)^{t_R + \tau_k} \cdot R \}$$

where *R* now represents the citations obtained by following the optimal submission schedule following *j* and *k*. Then the decision to start with *j* before *k* is given by $C_{jk} > C_{kj}$. That is,

$$\alpha_j\lambda_j(T-\tau_j) + (1-\alpha_j)(1-s)^{t_R+\tau_j}\{\alpha_k\lambda_k(T-\tau_j-t_R-\tau_k) + (1-\alpha_k)(1-s)^{t_R+\tau_k}\cdot R\}$$
$$\geq \alpha_k\lambda_k(T-\tau_k) + (1-\alpha_k)(1-s)^{t_R+\tau_k}\{\alpha_j\lambda_j(T-\tau_k-t_R-\tau_j) + (1-\alpha_j)(1-s)^{t_R+\tau_j}\cdot R\}$$

Noting that the terms involving R cancel, we can simplify to obtain criterion (2) in the main text. Using (2), we can compare journals pairwise to determine the citation-maximizing submission decision.

To obtain our value index (3) note that if $\frac{T-\tau_j-\tau_k-t_R}{T-\tau_j} = 1 - \frac{\tau_k+t_R}{T-\tau_j} \approx 1-\epsilon$, then to first order, (2) reduces to

$$\frac{\alpha_j\lambda_j(1-\tau_j/T)}{1-(1-\frac{\tau_j}{T}-\frac{t_R}{T})(1-\alpha_j)(1-s)^{t_R+\tau_j}} \geq \frac{\alpha_k\lambda_k(1-\tau_k/T)}{1-(1-\frac{\tau_k}{T}-\frac{t_R}{T})(1-\alpha_k)(1-s)^{t_R+\tau_k}}$$

Since the terms for each journal are now separable, we can score them independently using (3).
