# Supplementary material for: Where Should I Send It? Optimizing the Submission Decision Process
Source: PLoS One. 2015 Jan 23;10(1):e0115451. doi: 10.1371/journal.pone.0115451 (PMC4304711; doi:10.1371/journal.pone.0115451)
Supplement: S1 Table — (PDF) [file pone.0115451.s009.pdf]

Table S1

Data used in the analysis.

Journal	Acceptance rate	Sub.-to-dec. time (d)	2012 IF
Agriculture, Ecosystems and Environment	0.14	51.1	2.859
American Midland Naturalist	0.41	227.5	0.667
American Naturalist	0.22	46	4.552
Animal Conservation	0.182	31.8	2.692
Annales Zoologici Fennici	0.5	135	1.211
Aquatic Ecology	0.154	30.9	1.378
Basic and Applied Ecology	0.313	45.4	2.696
Behavioral Ecology	0.36	43	3.216
Biology Letters	0.212	22	3.348
Biotropica	0.42	20	2.351
Community Ecology	0.396	90	1.623
Ecography	0.25	90	5.124
Ecological Economics	0.203	52	2.855
Ecology Letters	0.124	32.7	17.949
Ecological Modelling	0.36	222	2.069
Ecological Monographs	0.27	28.1	8.085
Ecological Research	0.25	46.4	1.552
Ecology	0.26	48	5.175
Ecology and Society	0.45	105	2.831
Ecosystems	0.2	42	3.165
European Journal of Soil Biology	0.22	84	1.838
Evolution	0.285	44.5	4.864
Evolutionary Ecology	0.274	20.2	2.407
Functional Ecology	0.14	32.6	4.861
Global Change Biology	0.2	45	6.91
ISME Journal	0.24	45	8.951
Israel Journal of Ecology & Evolution	0.1	42	0.444
Journal of Animal Ecology	0.168	33.4	4.841
Journal of Applied Ecology	0.17	39.6	4.74
Journal of Ecology	0.201	29.5	5.431
Journal of Evolutionary Biology	0.34	27	3.479
Journal of the North American	0.44	67	2.957

Benthological Society			
Journal of Soil and Water Conservation	0.25	70	1.722
Landscape and Urban Planning	0.21	117.6	2.314
Marine Biology Research	0.35	56	0.962
Marine Ecology Progress Series	0.45	60	2.546
Microbial Ecology	0.321	72.8	3.277
Molecular Ecology	0.27	35	6.275
Molecular Ecology Resources	0.35	36	7.432
Natural Areas Journal	0.58	28	0.707
Northeastern Naturalist	0.7	90	0.362
Northwest Science	0.7	70.3	0.512
Oikos	0.17	50	3.322
Paleobiology	0.367	90	2.757
Pedobiologia	0.3	72.1	1.69
PLoS ONE	0.69	33.4	4.092
Polish Journal of Ecology	0.6	365	0.503
Polar Biology	0.53	128	2.006
Polar Record	0.745	30	0.981
Polar Research	0.67	70	1.622
Population Ecology	0.372	41.4	1.923
Proceedings of the National Academy of Sciences USA	0.17	41	9.737
Rangeland Journal	0.407	52.2	1.276
Restoration Ecology	0.25	32	1.934
Revue d'Ecologie	0.3	212.5	0.278
Russian Journal of Ecology	0.33	450	0.236
Science	0.07	84	31.027
Southeastern Naturalist	0.7	90	0.344
Texas Journal of Science	0.778	137.9	0.113
Western North American Naturalist	0.75	129	0.366
Wildlife Monographs	0.346	182	2.833
