# Supplementary material for: Where Should I Send It? Optimizing the Submission Decision Process
Source: PLoS One. 2015 Jan 23;10(1):e0115451. doi: 10.1371/journal.pone.0115451 (PMC4304711; doi:10.1371/journal.pone.0115451)
Supplement: S1 Text — (PDF) [file pone.0115451.s010.pdf]

Text S1

Editors who contributed data

Jürg Fuhrer (*Agriculture, Ecosystems and Environment*), Patricia Morse (*American Naturalist*), Dawn Denton (*American Midland Naturalist*), Elina Rantanen (*Animal Conservation*), Krzysztof Raciborski (*Annales Zoologici Fennici*), Jennifer Jongsma (*Annual Review of Ecology, Evolution and Systematics*), Piet Spaak (*Aquatic Ecology*), Klaus Hövemeyer, Teja Tscharntke (*Basic and Applied Ecology*), Leigh Simmons (*Behavioral Ecology*), Brian Charlesworth, Charlotte Wray (*Biology Letters*), Emilio Bruna (*Biotropica*), János Podani (*Community Ecology*), Maria Persson (*Ecography*), Anne Aitken (*Ecological Economics*), Nathalie Espuno, Marcel Holyoak (*Ecology Letters*), Brian Fath (*Ecological Modelling*), Aaron Ellison (*Ecological Monographs*), Atsushi Kume (*Ecological Research*), Adele Mullie (*Ecology and Society*), Anne Marie Whelan (*Ecology*), Suzann McClenahan (*Ecosystems*), Christoph Tebbe (*European Journal of Soil Biology*), John Endler (*Evolutionary Ecology*), Phaedra Cress, Ruth Shaw (*Evolution*), Jennifer Meyer (*Functional Ecology*), Rhea Kressman (*Global Change Biology*), Sarash de Wilde (*ISME Journal*), Leon Blaustein (*Israel Journal of Ecology & Evolution*), Peter Livermore, Kenneth Wilson (*Journal of Animal Ecology*), Andrea Baier (*Journal of Applied Ecology*), Andrea Baier, David Gibson (*Journal of Ecology*), Lianne Baker (*Journal of Evolutionary Biology*), Pamela Silver (*Journal of the North American Benthological Society*), Annie Binder, Jorge Delgado (*Journal of Soil and Water Conservation*), Paul Gobster (*Landscape and Urban Planning*), Franz Uiblein (*Marine Biology Research*), Matthias Seaman (*Marine Ecology Progress Series*), Karen Nelson, Zachary Romano (*Microbial Ecology*), Loren Rieseberg, Tim Vines (*Molecular Ecology, Molecular Ecology Resources*), Ron Hiebert (*Natural Areas Journal*), Keith Goldfarb (*Northeastern

Naturalist), Nancy Grunewald, Jeremy Littell (*Northwest Science*), Åsa Langefors (*Oikos*), Julia Tejada-Lara (*Paleobiology*), Silke Guddat (*Pedobiologia*), Anna Hillbricht-Ilkowska (*Polish Journal of Ecology*), Dieter Piepenburg (*Polar Biology*), Ian Stone (*Polar Record*), Helle Goldman (*Polar Research*), Kazunori Sato (*Population Ecology*), Etta Kavanagh (*Proceedings of the National Academy of Sciences USA*), Chris Anderson (*Rangeland Journal*), Richard Hobbs, Susan Yates (*Restoration Ecology*), Christian Erard (*Revue d'Ecologie*), Vera Pozolotina (*Russian Journal of Ecology*), Janene Auger (*Western North American Naturalist*), Eric Hellgren (*Wildlife Monographs*).
